# Bridging the gap: a systematic literature review and meta-analysis on the management of rectal wall defect after transanal excision

**DOI:** 10.1007/s10151-026-03342-4

**Published:** 2026-05-16

**Authors:** S. Vaghiri, E. Gorgun, H. Kessler, W. T. Knoefel, D. Prassas

**Affiliations:** 1 Department of Surgery (A), Heinrich-Heine-University, Medical Faculty and University Hospital Duesseldorf, Moorenstr. 5, 40225 Duesseldorf, Germany; 2 Department of Colorectal Surgery, Digestive Disease and Surgery Institute, Cleveland Clinic, 9500 Euclid Avenue, Cleveland, OH 44195 USA; 3 Department of Surgery, Katholisches Klinikum Essen, Philippusstift, Teaching Hospital of Duisburg-Essen University, Huelsmannstr. 17, 45355 Essen, Germany

**Keywords:** Transanal surgery, Rectal excision, Defect closure, Postoperative morbidity, TEM, TAMIS

## Abstract

**Background:**

Transanal resection techniques have gained considerable importance in the treatment of benign and malignant rectal neoplasms. However, there is no definitive consensus on whether the rectal defect should be closed or left open after excision. We sought to provide an updated pooled analysis of the management of rectal wall defect after transanal excision.

**Methods:**

In accordance with PRISMA and Cochrane guidelines, this meta-analysis was performed using the PubMed (MEDLINE), Cochrane Central Register of Controlled Trials, and Google Scholar databases to identify studies comparing perioperative outcomes after rectal defect closure versus leaving the defect open following full-thickness (FT) excision. Odds ratios (ORs) and standardized mean differences (SMDs) with 95% confidence intervals (CIs) were calculated. Heterogeneity was assessed using Cochrane's *Q* test. Risk of bias and certainty of evidence were judged by ROBINS-I and GRADE, respectively.

**Results:**

Six comparative studies meeting the inclusion criteria were included in the final analysis. Closing the rectal defect was associated with significantly reduced rectal bleeding (OR = 0.57, 95% CI: 0.35–0.94, *p* = 0.03; *I*^2^ = 26%) and re-admission rates (OR = 0.34, 95% CI: 0.16–0.76, *p* = 0.008; I^2^ = 0%) compared with the open group, while other outcomes were not significantly different. A prolonged operative time was noted when the rectal wall defect was sutured (SMD = 0.15, 95% CI: 0.03–0.28, *p* = 0.02; I^2^ = 44%).

**Conclusions:**

The present analysis revealed that both approaches are safe and technically feasible; however, closing the rectal wall defect after FT excision of rectal neoplasms was associated with lower bleeding and re-admission rates. Nevertheless, randomized studies with homogeneous protocols and consistent long-term outcome data are still needed to provide definitive answers.

**Supplementary Information:**

The online version contains supplementary material available at 10.1007/s10151-026-03342-4.

## Introduction

Transanal excision of rectal neoplasms of the lower rectum has traditionally been performed via direct visualization of the lesion by using an anal retractor, such as the Parks retractor, and other conventional surgical apparatus. The concept of transanal endoscopic microsurgery (TEM) was first introduced by Buess four decades ago as an alternative to traditional local resection, greatly enhancing exposure and allowing access to more proximal rectal pathologies [1]. With the advent of minimally invasive surgery and laparoscopy, the concept of transanal minimally invasive surgery (TAMIS) emerged as a combination of TEM and a single-port approach and was  first reported in 2010 [2]. All these advanced endoluminal platforms represented a giant leap forward, eliminating structural problems of the traditional Parks transanal excision and enabling improved quality of the resected specimens [3]. Nevertheless, a technically demanding step is common to all excisional methods: closure of the rectal defect. Closure is mandatory for lesions above the peritoneal reflection, but not for those located in the lower two-thirds of the rectum. In the literature, there is no clear consensus on how the rectal defect should be managed, as underlined by conflicting results and conclusions [4–6].

Therefore, this meta-analysis aims to clarify whether rectal defect closure is necessary and to provide colorectal surgeons with evidence to guide this everyday clinical decision.

## Materials and methods

This systematic review and meta-analysis was conducted in accordance with the current PRISMA (Preferred Reporting Items for Systematic Reviews and Meta-Analyses) statement and the latest version of the Cochrane Handbook for Systematic Reviews of Interventions [7, 8]. The recommendations of both references were strictly adhered to at every step of the analysis. This work was registered in PROSPERO under the ID number CRD420251160705.

### Search strategy

Two independent authors (S.V. and D.P.) conducted the systematic literature search by screening studies in the PubMed (MEDLINE), the Cochrane Central Register of Controlled Trials, and Google Scholar databases until September 2025. The following medical keywords were combined using the Boolean operators AND or OR: [(defect closure) AND (TEM OR TAMIS OR transanal OR rectal)]. No time restrictions were imposed to ensure completeness and adequacy of source identification. The literature search was limited to English-language papers. Additionally, the reference lists of all retrieved articles—including comparative studies, systematic reviews, case reports, editorials, technical notes, or conference contributions, and abstracts—were comprehensively reviewed for further potentially relevant citations. Disagreements were resolved through discussion and consensus or, if necessary, by consultation with a third author (E.G.).

### Inclusion and exclusion criteria

The PICO framework was used to define the subject of this study and to structure it as follows:

P (Population): Patients undergoing full-thickness (FT) local excision of benign or malignant rectal neoplasms using transanal resection, TEMS, or TAMIS.

I (Intervention): Closing the defect after excision.

C (Comparison): Non-closure of the excision site.

O (Outcomes): Local infection (at the excision site) and rectal bleeding served as the primary outcomes. Operative time (min), length of hospital stay (LOS) in days, urinary dysfunction, postoperative rectal stricture/stenosis, re-admission and re-intervention rates, and overall postoperative morbidity were secondary outcomes.

Accordingly, all comparative studies (e.g., randomized controlled trials [RCTs], nonrandomized clinical trials, and nonrandomized observational studies) reporting perioperative outcomes of defect closure at the excision site versus leaving the defect open during TEMS, TAMIS, or transanal excision for rectal neoplasms were considered eligible. The following exclusion criteria were applied: a) ineligible sources such as non-comparative studies, case reports, technical reports, editorials, narrative reviews, and grey literature; b) duplicate or overlapping articles by the same authors or institutions; c) nonadult patients (aged < 18 years); d) inability to calculate odds ratios (ORs) or standardized mean differences (SMDs) with 95% confidence intervals (CIs) for the reported outcomes of interest; e) short-term follow-up (< 30 days); and f) failure to report at least one of the primary outcomes of interest. Discrepancies in study selection were resolved either by consensus or by consultation with an independent third author (W.T.K.).

### Data extraction

Two authors (S.V. and D.P.) independently collected all available and relevant data from studies meeting the inclusion criteria. The following information was extracted: a) study characteristics (year and country of publication, name of authors, enrollment period, study design, number of patients in each group); b) patient demographics, disease characteristics, and tumor localization, as well as perioperative patient preparation and management; c) type and platform of resection, information on defect closure and devices applied, and d) short- and long-term postoperative outcomes. Where necessary,consensus was reached during the data extraction process by consulting a third author (H.K.).

### Risk of bias and certainty assessment

The ROBINS-I tool [9] was used to assess the risk of bias in all nonrandomized studies, following the Cochrane Handbook for Systematic Reviews of Interventions. Two authors (S.V. and E.G.) independently evaluated each study across seven well-established bias domains and finally categorized the risk of bias as low, moderate, serious, or critical. The GRADE (The Grading of Recommendations, Assessment, Development, and Evaluation) criteria [10] were applied to assess the robustness and certainty of evidence for significant outcomes. Final consensus was achieved and approved by all authors.

### Statistical analyses

**Fig. 1:**
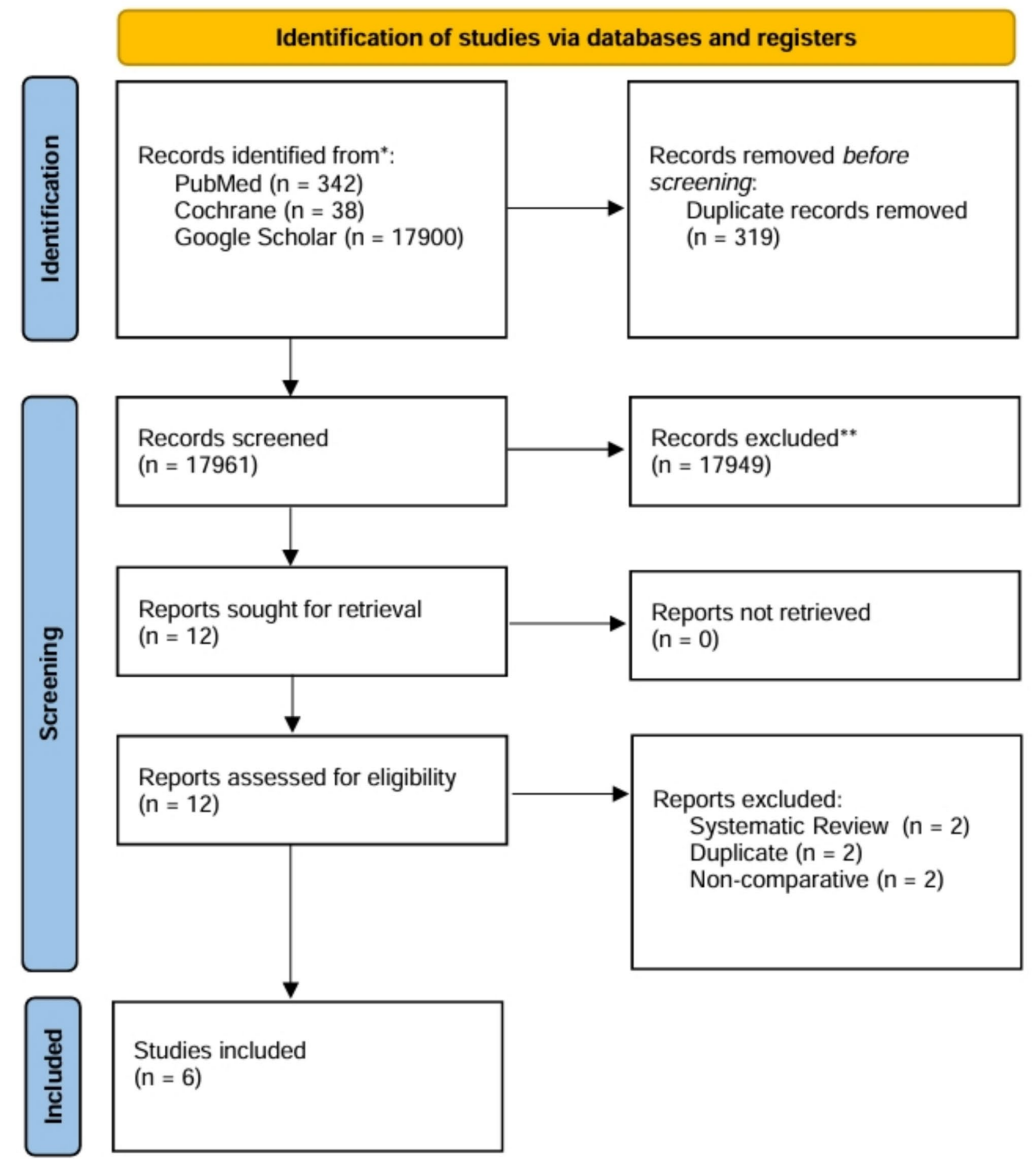
PRISMA flowchart of study search and selection

Statistical analysis was performed using RevMan software (version 5.3; Copenhagen: The Nordic Cochrane Centre, The Cochrane Collaboration, 2014). Pairwise meta-analyses were conducted for outcomes of interest. For each primary and secondary outcome, summary treatment effect estimates with 95% confidence intervals (CIs) were presented. For dichotomous end points, odds ratios (ORs) were used as the effect measure and pooled for quantitative synthesis using the Cochrane Mantel–Haenszel method. Standardized mean differences (SMDs) were calculated to analyze continuous outcomes when applicable. Effect sizes of continuous outcomes were summarized using the inverse-variance method. Where necessary, medians and ranges were converted into means and standard deviations using the method proposed by Hozo et al. [11]. Fixed- or random-effects models were applied as appropriate to calculate summary estimates, according to standardized and recommended methodology. Using Cochrane's *Q* test (chi-squared test; χ^2^) and the measure of inconsistency (*I*^2^), the degree of heterogeneity was interpreted as follows: 0%-40% low (may not be important), 30%-60% moderate, 50%-90% substantial, and > 75% considerable heterogeneity [8]. For outcomes exhibiting substantial heterogeneity (I^2^ > 50%), the source of heterogeneity was explored by means of subgroup analysis (leave-one-out and/or post-hoc grouping). Furthermore, to minimize bias, subgroup analyses were conducted for specific outcomes (e.g., urinary dysfunction and rectal stenosis/stricture) based on the follow-up period (short-term: < 30 days versus long-term: > 12 months). These analyses were not prespecified in the protocol and should therefore be considered exploratory. Owing to the limited number of included studies, publication bias tests (e.g., Egger’s test) and funnel plots were not performed. A *p*-value < 0.05 was the threshold of statistical significance (Fig. 1).

## Results

### Study-and patient characteristics

A total of 17,961 records were screened after the database research. Based on the predefined eligibility criteria, six studies were included in the final analysis [12–17]. Three studies originated from Europe [12, 16, 17], one from Japan [13], and two from North America [14, 15]. Notably, the RCT by Ramirez et al. [4] was not included, because the study by Gracia et al. [17] conducted at the same institution, provided a more recent analysis with a substantially larger sample size and a significantly longer follow-up period. The overall study population comprised 1,253 cases of local excision of rectal tumors, divided into the open (*n* = 603) and closure (*n* = 650) groups. The study enrollment periods across all studies ranged from 1997 to 2019. All studies included both benign and malignant entities [12–17]. Final pathology in four studies revealed more advanced tumor stages (e.g., pT2-3) [13, 15–17]. The TAMIS platform was used exclusively in one study [12], while two studies applied both TAMIS and TEM or the Parks retractor for tumor excision [13, 15]. In the studies by Brown et al. [14], Altaf et al. [16], and Gracia et al. [17], TEM was the preferred approach for tumor removal. All patients received full preoperative bowel preparation, except in the study by Lee et al. [15], in which only TAMIS patients underwent bowel preparation. Prophylactic antibiotics were administered in all studies [12–17]. The mean distance from the anal verge ranged from 4.3 cm in Noura et al. [13] to 9.6 cm in Gracia et al. [17]. No direct data regarding defect size were provided; however, postexcisional defects can be assumed  not to have differed substantially among the compared groups and studies, as tumor size and localization were comparable in all six studies, with the sole exception of Brown et al. [14], who reported a significantly more proximal tumor localization in the closure group. Short-term (30-days) [14, 15] and long-term (> 12 months) [12, 13, 16, 17] follow-up data were provided. The main study and patient characteristics are summarized in Tables 1 and 2.

**Table 1:** Study characteristics and technical considerations

Author	Origin	Study period	Study design	Total sample size	Platform of excision	Type of resection	Closing technique	Full bowel preparation	Prophylactic antibiotics	Inclusion criteria	Follow-up period
Hahnloser et al. [12]	Switzerland	Dec 2009–Jul 2012	Prospective, multicenter	75	TAMIS/SILS Port	FT	Interrupted or running suture Vicryl 3–0 or V-lock 3–0	All patients	All patients	Low grade rectal adenoma, rectal adenocarcinoma, carcinoid tumor	median 385 days (67–884)
Noura et al [13]	Japan	Jan 2004–Mar 2015	Retrospective, single-center	43	Parks’ retractor TAMIS/GelPoint®	FT	NA	All patients	All patients	Rectal neoplasm max. 3 cm ⌀, cT1 or 2, cN0	> 12 months
Brown et al [14]	Canada	2007–2014	Prospective, single-center	341	TEM	FT	Running suture 2–0 PDS and suture clip forceps	All patients	All patients	Age > 18 years, FT TEM, Adenoma, Carcinoma, Carcinoid, GIST	30-days
Lee et al [15]	USA	2004–2016	Prospective, propensity scored, multicenter	991	TEM TAMIS/GelPoint®	FT 593 PT 398	3–0 absorbable sutures	Only TAMIS	All patients	Local rectal excision for malignant or benign neoplasm (early and advanced stages T0–T3)	30-days
Altaf et al [16]	UK	Jan 2012–Dec 2019	Prospective, single-center	170	TEM/TEO	FT	Vicryl 3–0 or V-lock	All patients	All patients	Benign and malignant rectal neoplasms	> 12 months
Gracia et al [17]	Spain	1997–2019	Retrospective, single-center	404	TEM Transanal (Parks’ retractor)	FT	Running suture 3.0 monofilament	All patients	All patients	Age > 18 years, only transanal FT excision of benign sessile adenomas or early rectal cancer	mean 58 months (12–96)

*FT* Full thickness, *PT* Partial thickness, *SILS* Single incision laparoscopic surgery, *TAMIS* Transanal minimally invasive surgery, *TEM* Transanal endoscopic microsurgery, *TEO* Transanal endoscopic operation, *NA* Not available

**Table 2:** Patient demographics and tumor characteristics

Author	Groups	Number of patients	Gender ratio (M/F)	Age (years) mean ± SD	Tumor location	Distance from anal verge (cm) mean ± SD	Specimen size (cm) mean ± SD	Resection margin involved	Platform of excision
Hahnloser et al. [12]	Closed	40	51/24	67.3 ± 14.9	Anterior 11	9.1 ± 2.9	1404 ± 1078†	NA	TAMIS/SILS 40
	Open	35			Anterior 2	9.3 ± 1.7	1218 ± 914		TAMIS/SILS 35
Noura et al. [13]	Closed	21	11/10	59.6 ± 8.6	Anterior 8 Posterior 8 Lateral 5	4.3 ± 1.6	2.2 ± 0.8	3	Parks’ retractor 21 TAMIS/GelPOINT® 0
	Open	22	11/11	64.7 ± 7.8	Anterior 6 Posterior 11 Lateral 5	4.5 ± 1.8	2.6 ± 1.2		Parks’ retractor 14 TAMIS/GelPOINT® 8
Brown et al. [14]	Closed	236	141/95	67 (24–99)*	Anterior 44 Posterior 65 Lateral 127	7.9 (1–15)*	3.1 (0.1–9.0)*	NA	TEM 236
	Open	105	66/39	69 (22–94)	Anterior 28 Posterior 31 Lateral 46	6.9 (1–15)	3.5 (0.3–9.0)		TEM 105
Lee et al. [15]	Closed	110	73/37	65.8 ± 11.3	Anterior 21	6.6 ± 2.6	3.3 ± 1.4	8	TEM 55 TAMIS 55
	Open	110	77/33	65.4 ± 14.8	Anterior 16	6.4 ± 3.0	3.4 ± 1.6	7	TEM 100 TAMIS 10
Altaf et al. [16]	Closed	100	61/32	70 (47–89)*	Anterior 25 Posterior 39 Circumferential 1 Lateral 36	5 (2–12)*	3.0 (2–6.5)*	NA	TEM 100
	Open	70	58.19	72 (27–81)	Anterior 19 Posterior 26 Circumferential 2 Lateral 22	6 (1–8)	3.5 (1–10)		TEM 70
Gracia et al. [17]	Closed	143	72/71	68.7 ± 11.9	Anterior 34 Posterior 63 Circumferential 4 Lateral 42	9.65 ± 3.44	3.61 ± 1.34	5	TEM 126 Transanal 17
	Open	261	167/94	67.6 ± 11.9	Anterior 69 Posterior 132 Circumferential 13 Lateral 47	9.51 ± 4.08	3.68 ± 1.67	15	TEM 238 Transanal 23

*SILS* Single incision laparoscopic surgery, *TAMIS* Transanal minimally invasive surgery, *TEM* Transanal endoscopic microsurgery, *NA* Not available, * median (range), † tumor surface mm^2^

### Risk of bias evaluation

**Fig. 2:**
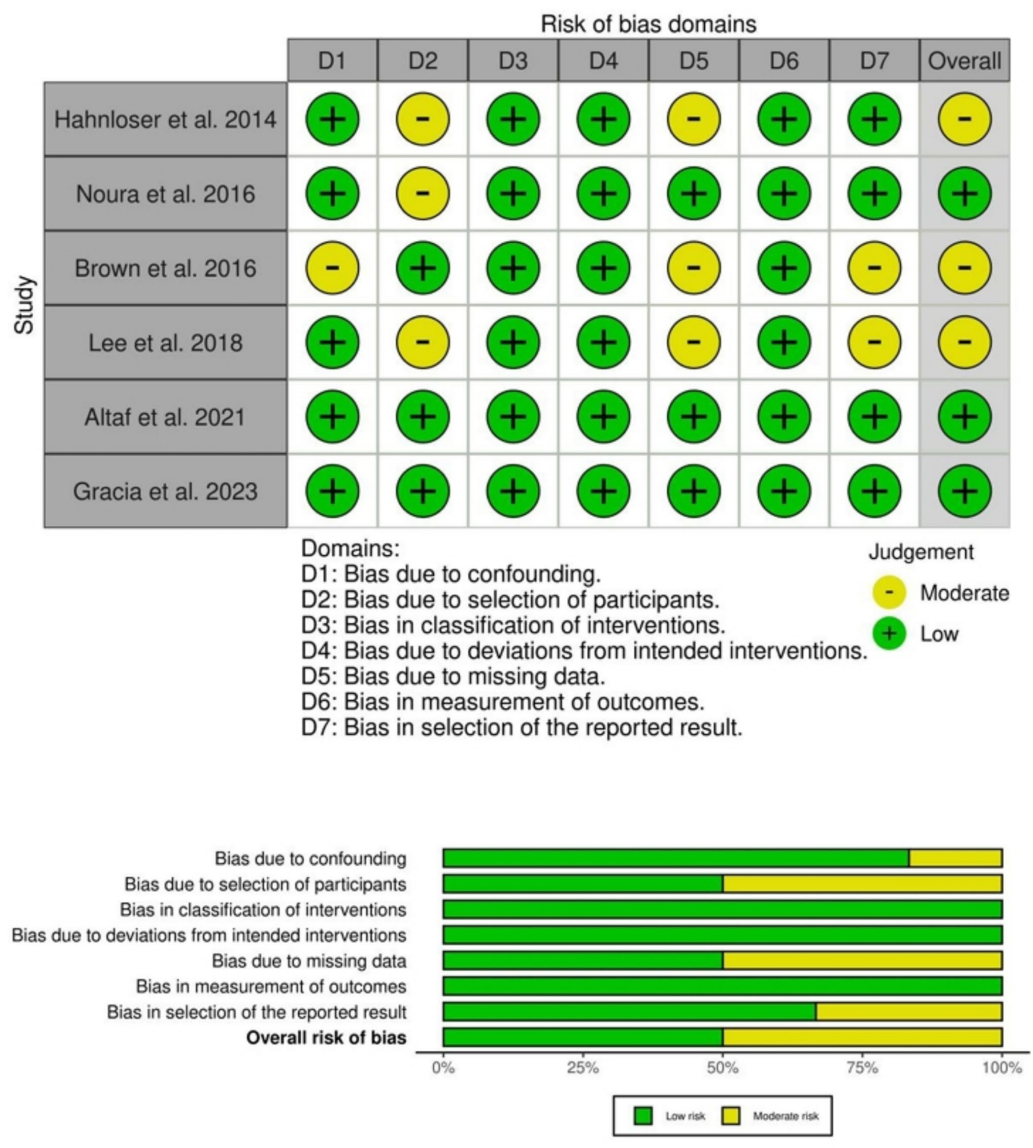
ROBINS-I risk of bias summary

The overall risk of bias was assessed as low in three studies [13, 16, 17] and moderate in the remaining three included studies [12, 14, 15]. The detailed risk-of-bias evaluation for each domain is shown in Fig. 2. All included studies had an observational, non-randomized design [12–17]. Propensity score-matched analysis was conducted in the study by Lee et al. [15]. Other factors contributing to an increased risk of bias included: a) selection bias in either group; b) significant differences in platform choice between the open and closure groups [13, 15]; c) varying follow-up durations; d) missing data for some outcomes; and e) heterogeneous measurement of outcomes (e.g., incontinence).

### Main outcomes

#### Local infection

**Fig. 3:**
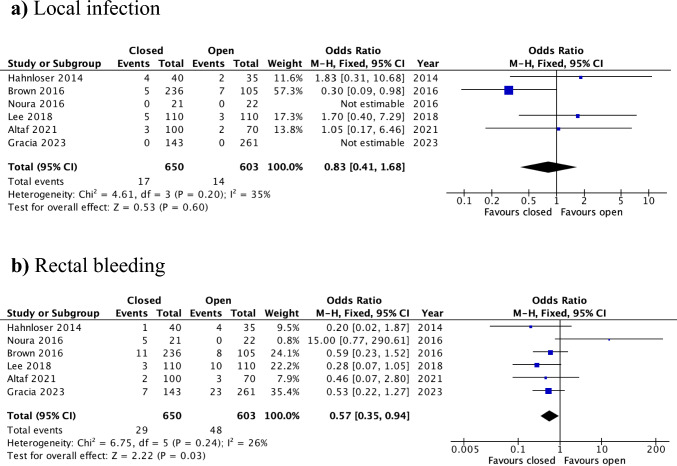
Forest plots for the main outcomes, closure versus open: **a** local infection, **b** rectal bleeding

The rate of local infection was reported in all studies, encompassing 650 patients in the closed group and 603 patients in the open-defect group [12–17]. There was no significant difference in the incidence of local infection between the two groups, with low heterogeneity (OR = 0.83, 95% CI: 0.41–1.68, *p* = 0.60; I^2^ = 35%) (Fig. 3a).

#### Rectal bleeding

Postoperative rectal bleeding was reported in all studies, with a total of 1,253 patients (open: *n* = 603; closed: *n* = 650) [12–17]. Pooled analysis revealed that the rate of postoperative rectal bleeding was significantly lower in the closed-defect group compared with leaving the defect open (OR = 0.57, 95% CI: 0.35–0.94, *p* = 0.03; I^2^ = 26%) (Fig. 3b). GRADE assessment confirmed a moderate level of certainty of evidence (Supplementary Table 1).

### Secondary outcomes

#### Operative time

**Fig. 4:**
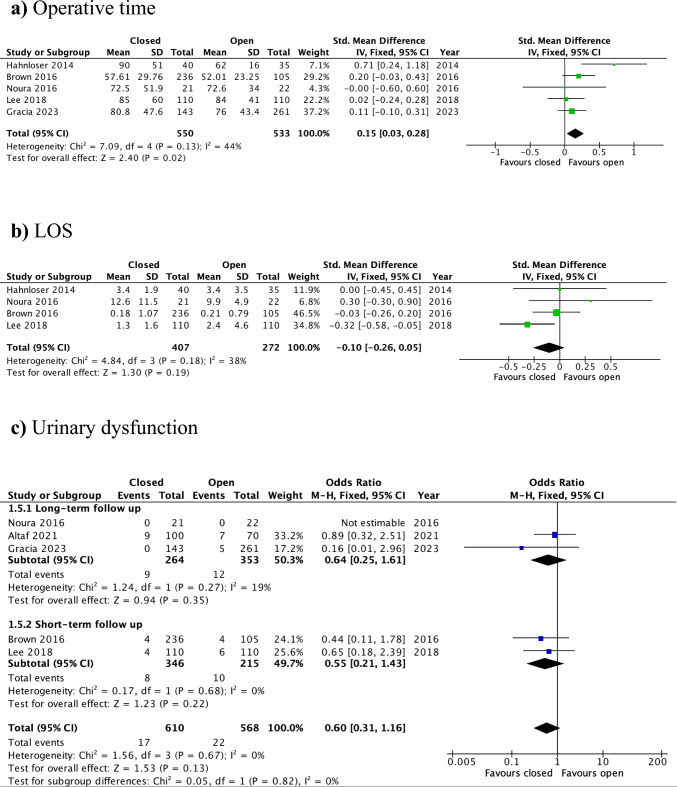

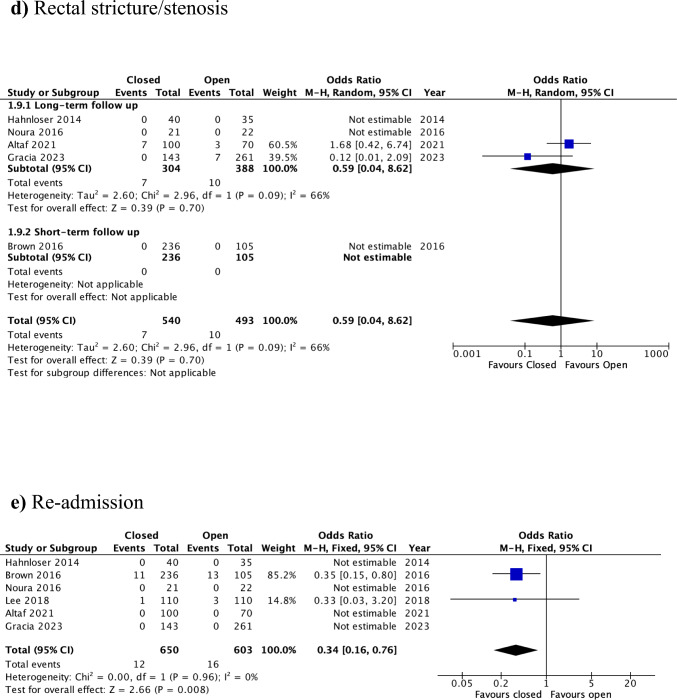

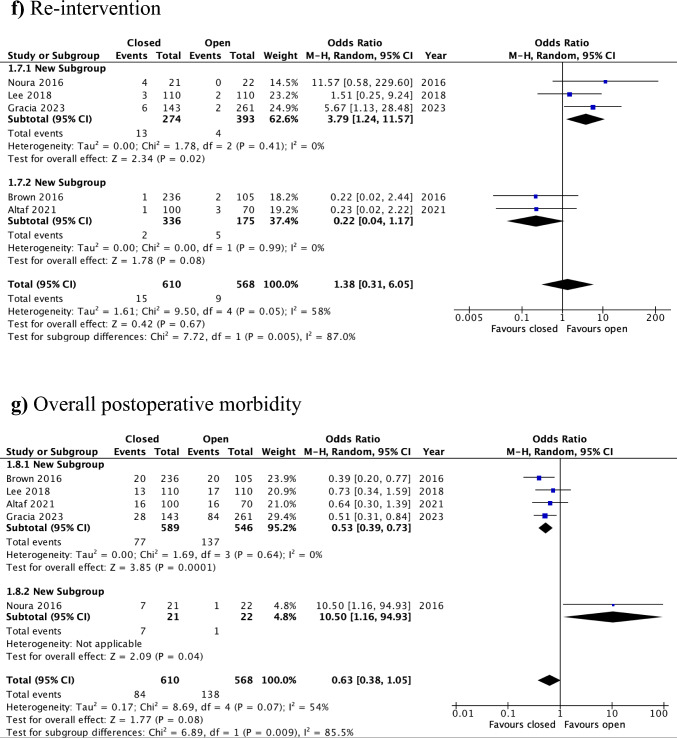
Forest plots for the secondary outcomes, closure versus open: **a** operative time, **b** LOS, **c** urinary dysfunction, **d** rectal stricture/stenosis, **e** re-admission, **f** re-intervention, **g** overall postoperative morbidity

The duration of the procedure was reported in five studies, encompassing 1,083 patients (open: *n* = 533; closed: *n* = 550) [12–15, 17]. Operative time was significantly shorter [by a mean difference of 6.35 min (95%CI: 1.93–10.78)] when the defect was left open compared with the closure group (SMD = 0.15, 95% CI: 0.03–0.28, *p* = 0.02; I^2^ = 44%) (Fig. 4a). The certainty of evidence was judged to be moderate (Supplementary Table 1).

#### Length of hospital stay (LOS)

LOS was reported in four studies, including 407 patients in the closed group and 272 patients in the open group [12–15]. The pooled analysis demonstrated no significant difference in the duration of postoperative hospital stay between the two groups (SMD = −0.10, 95% CI: −0.26 to 0.05, *p* = 0.19; *I*^2^ = 38%) (Fig. 4b).

#### Urinary dysfunction

In five studies [13–17], urinary dysfunction was observed in 39 patients across the overall cohort. The rate of urinary dysfunction was comparable between the open and closure groups (OR = 0.60, 95% CI: 0.31–1.16, *p* = 0.13; *I*^2^ = 0%). Additionally, a subgroup analysis was performed on the basis of the reported follow-up duration in each included study. No statistically significant differences in the occurrence of urinary dysfunction were observed between the two cohorts in either the short-term or long-term follow-up subgroups (Fig. 4c).

#### Rectal stricture/stenosis

Postoperative rectal stricture/stenosis was reported in five studies [12–14, 16, 17], with 17 confirmed events among 574 patients for this outcome, as reported by two authors [16, 17]. There was no significant difference in the incidence of rectal stricture/stenosis between the two groups, either in the overall analysis or in the subgroup analysis based on the follow-up interval (OR = 0.59, 95% CI: 0.04–8.62, *p* = 0.7; *I*^2^ = 66%) (Fig. 4d).

#### Re-admission

The re-admission rate was documented in all studies, with 1,253 included patients (open: *n* = 603; closed: *n* = 650) [12–17]. Closure of the rectal defect was associated with a significantly lower rate of re-admission compared with leaving the defect open (OR = 0.34, 95% CI: 0.16–0.76, *p* = 0.008; I^2^ = 0%) (Fig. 4e). The GRADE judgement for this outcome was low (Supplementary Table 1).

#### Re-intervention

The need for re-intervention was reported in five studies, including a total of 1,178 patients (open: *n* = 568; closed: *n* = 610) [13–17]. Overall, there was no significant difference between the two approaches in re-intervention rates (OR = 1.38, 95% CI: 0.31–6.05, *p* = 0.67; I^2^ = 58%). The source of increased heterogeneity was identified in the studies by Brown et al. [14] and Altaf et al. [16], which formed a homogeneous subgroup (*I*^2^ = 0) and showed a tendency toward higher re-intervention rates in the open group (OR = 0.22, 95% CI: 0.04–1.17, *p* = 0.08). In contrast, subgroup analysis of the remaining three studies [13, 15, 17] revealed significantly lower re-intervention rates in the open group than in the closed-defect cohort (OR = 3.79, 95% CI: 1.24–11.57, *p* = 0.02; I^2^ = 0%) (Fig. 4f). The certainty of evidence was low (Supplementary Table 1).

#### Overall postoperative morbidity

Five studies, including a total of 1,178 patients, reported on overall postoperative morbidity [13–17]. There was no significant difference in overall postoperative morbidity between the open (*n* = 568) and closed (*n* = 610) groups (OR = 0.63, 95% CI: 0.38–1.05, *p* = 0.08; I^2^ = 54%). The study by Noura et al., which included only 43 cases in total, contributed to substantial heterogeneity [13]. Subsequent subgroup analysis of the remaining four studies [14–17] demonstrated a significantly lower rate of overall postoperative morbidity in the closed-defect group compared with the open-defect group (OR = 0.53, 95% CI: 0.39–0.73, *p* = 0.0001; I^2^ = 0%) (Fig. 4 g). This outcome was assigned a low certainty of evidence according to GRADE (Supplementary Table 1).

## Discussion

Local excision of benign broad-based polypoid lesions and selected malignancies has established itself as a safe and effective practice that avoids the sequelae of radical major surgery [18], with even more options being available, including advanced endoscopic resections such as Endoscopic Mucosal Resection (EMR) and Endoscopic Submucosal Dissection (ESD) [19, 20]. Indications for local excision are currently expanding beyond early cancers, with increasing data supporting this approach. Recently, a randomized multicenter clinical trial demonstrated non-inferior outcomes with transanal excision for T2 and T3ab, N0, M0 rectal cancers compared with standard radical surgical treatment in terms of local recurrence, with similar results for distant recurrence (DR), overall survival (OS), and disease-free survival (DFS) [21]. In the ever-evolving landscape of rectal cancer treatment, local resection of rectal lesions plays a pivotal role in organ-preserving therapeutic regimens and is  becoming increasingly relevant than ever with an upward trend [22]. For every colorectal surgeon performing these excisions, regardless of the platform they use, the question of whether the resulting rectal defect should be closed in this otherwise straightforward procedure remains largely unanswered. To date, two meta-analyses have addressed this topic. The first suggested that there is no difference between closure and non-closure of the wall defect after local excision [5], whereas the second, and more recent meta-analysis reported a reduced incidence of postoperative bleeding associated with leaving the defect open [6]. In light of this, we conducted an updated meta-analysis of studies comparing closure and non-closure of the rectal defect. Six studies were included, encompassing all transanal local excisional approaches of rectal neoplasia [12–17]. We found no significant difference between the two groups with regard to local infection. All studies except one [14] reported lower or equal rates of local infection findings in the open group. The contradictory findings of Brown et al. [14] could be attributed to a combination of selection bias and a rather liberal definition of the outcome of local infection as “peritonitis or pelvic pain and either fever (> 37.9 °C) or white blood cell (WBC) > 11 × 109 c/L or clinician diagnosed postoperative infection”. It is also noted that the most experienced surgeon performed the majority of the closure cases. This is also reflected in the significantly lower overall morbidity of this subgroup (8.4% versus 19%, *p* = 0.003). Clinically, healing of the postoperative intraluminal wound is complete in 95% of sutured cases and 84% of unsutured cases four weeks after surgery [12]. Suturing the rectal defect in a tension-free manner is, in many instances, not feasible. Hahnloser et al. [12] reported that in almost one out of three operated patients, the rectal defect resulting from excision were considered non-closable. The question that arises is whether this subgroup of patients should be left open rather than sutured under tension. Data on this specific subcategory of defects are, to the best of our knowledge, lacking. This finding is in line with data presented by Dulskas et al. [23], who suggest that 32% (*n* = 22) of patients’ rectal wall defects after TEM undergo dehiscence in the early postoperative period, without causing any clinically significant manifestation. More specifically, 8 of 22 patients (36.4%) with suture dehiscence had per-rectal bleeding or febrile temperature, without any need for intervention or treatment. In our meta-analysis, all six included studies reported on rectal bleeding [12–17]. Only two studies [13, 15] reported significantly different rates of rectal bleeding between the groups: Noura et al. [13] in favor of the open group (0% versus 23.8%, p = 0.02), and Lee et al. [15] with higher bleeding rates in the open group (9% versus 3%, *p* = 0.045). The remaining four studies [12, 14, 16, 17] all demonstrated a tendency toward fewer bleeding incidents when the defect was closed. This could be explained by the fact, that in the closure subgroup of Noura et al. [13], an electric cautery device was used exclusively for the resection instead of a vessel-sealing-device. Nevertheless, a clear benefit of defect closure was demonstrated (OR = 0.57, 95% CI: 0.35–0.94, *p* = 0.03; I^2^ = 26%). The same subgroup in Noura et al. [13] was also the source of heterogeneity in the meta-analysis of overall morbidity, as five of seven postoperative complications in the defect-closure group werepostoperative bleeding events directly attributed to the use of an electric cautery device. After exclusion of the above-mentioned biased result, we conclude that overall morbidity was significantly lower when the defect was closed (OR = 0.53, 95% CI: 0.39–0.73, *p* = 0.0001; I^2^ = 0%). Defect closure was also found to be beneficial in terms of reduced re-admission rates (OR = 0.34, 95% CI: 0.16–0.76, *p* = 0.008; I^2^ = 0%). All six included studies [12–17] reported this outcome, with two documenting actual re-admissions, both in favor of the closure group [14, 15]. Main reasons for re-admission in the series by Brown et al. [14] were bleeding, followed by urinary retention. The authors stated that they adopted a policy of discharging elderly patients at risk of urinary retention with a Foley catheter, which was removed 24–48 h later, thereby practically eliminating urinary retention as a reason for re-admission. Our analysis did not show any association between management of the rectal defect and urinary retention (OR = 0.60, 95% CI: 0.31–1.16, *p* = 0.13; *I*^2^ = 0%). Interestingly, this finding was confirmed in studies with both short-term [14, 15] and long-term [13, 16, 17] follow-up data.

As expected, when performed, defect closure results in a limited yet significant increase in operative time. The confined endorectal workspace leads to frequent instrument collisions, resulting in a challenging and time-consuming surgical step. Five of the six included studies [12, 14–17] reported their defect-closure technique, which consisted of either per interrupted or running suture. Other alternative methods of defect closure intended to shorten procedural time include the use of stapling devices [2] and extracorporeal suturing with implementation of a knot pusher [24]. To date, no data exist to show the superiority of one method over another. Intuitively, it is often hypothesized that primary narrowing of the rectal lumen through defect closure may place the patient at higher risk of rectal stenosis. Five of the included studies [12–14, 16, 17] reported on this outcome, with three [12–14] demonstrating no cases of stenosis at all. The two remaining studies reported contradictory findings. Gracia et al. [17] noted rectal strictures in 7 of 261 patients (2.7%) exclusively in the open subgroup, whereas Altaf et al. [16] reported an incidence of stenosis in 3 of 70 patients (4%) in the open subgroup, as opposed to 7 of 100 (7%) in the closure group. However, no further treatment was needed, as the endoscopists were able to pass the scope through all of them. Unlike Gracia et al. [17], Altaf et al. [16] systematically categorized the sigmoidoscopic finding as completely healed, unhealed/sinus formation, and stricture/narrowing at the scar site. That being said, we cannot draw any conclusions regarding the severity and/or clinical relevance of the strictures reported in the series of Gracia et al. [17]. Essentially, the most interesting aspect of this outcome is its clinical impact on patients in terms of deterioration of continence resulting from reduced rectal wall redundancy and loss of sensory fibers. Altaf et al. [16] specifically investigated this issue, focusing on the severity of endoscopic pathological findings. A significant difference was observed only in the subgroup of patients with completely healed excisional sites, in whom closure of the rectal defect was associated with worsened function, but not in those with postoperative sinus or stricture formation. This is a surprising finding that should be further explored with long-term anorectal functional studies. No significant difference in postoperative anorectal function was noted in the three included studies that investigated it [12, 13, 17]. Notably, for lesions involving more than 50% of the rectal circumference, full-thickness excision may not be feasible, and closure techniques can lead to diminished reservoir capacity of the rectum, leading to poorer functional outcomes.

This meta-analysis has certain limitations. First, the number of eligible studies was relatively small, and there was a noticeable lack of randomized studies. In addition, heterogeneity arising from the use of different operative techniques may affect the comparability of results. Postoperative surveillance strategies, medical treatment protocols-which evolved even within the same study period-, and differences in follow-up duration also varied considerably. Another major limitation of the present analysis is the risk of confounding by indication and selection bias. In routine practice, defect closure is not randomly applied but depends on technical feasibility and surgeon judgment. Smaller, distally located, and technically less complex lesions are generally more amenable to closure, whereas large, proximally located, or technically challenging defects may be left open. Therefore, patients in the open group may systematically differ from those in the closure group. In addition, surgeon experience and platform-specific capabilities may influence both the likelihood of closure and clinical outcomes.

However, the present study provides a comprehensive and updated meta-analysis on this subject, providing novel robust evidence regarding the clinical value and necessity of the final step in FT excisions of rectal neoplasia.

## Conclusions

The present data suggest that, after transanal full-thickness excision of rectal neoplasia, both approaches are safe and technically feasible, with largely comparable short- and long-term outcomes. Owing to potential sources of bias, the results should be interpreted with caution. Future prospective and randomized studies with standardized reporting of lesion size, defect characteristics, and predefined criteria for defect closure focusing on consistent long-term functional outcomes, are warranted.

## Supplementary Information

Below is the link to the electronic supplementary material.

Supplementary file1 (DOCX 269 KB)

Supplementary file2 (DOCX 25 KB)

## Data Availability

The datasets used and/or analyzed during the current study are available from the corresponding author on reasonable request.
