# Supplementary material for: Bridging the gap: a systematic literature review and meta-analysis on the management of rectal wall defect after transanal excision
Source: Tech Coloproctol. 2026 May 16;30(1):65. doi: 10.1007/s10151-026-03342-4 (PMC13179188; doi:10.1007/s10151-026-03342-4)
Supplement: Supplementary file 2 — Supplementary file2 (DOCX 25 KB) [file 10151_2026_3342_MOESM2_ESM.docx]

| Table Suppl. 1 The GRADE Certainty assessment for the significant outcomes | | | | | | | | | | |
| --- | --- | --- | --- | --- | --- | --- | --- | --- | --- | --- |
| Outcomes | No. of studies | **No. of included patients** | | OR/SMD [95 % CI] | **Quality assessment** | | | | | Quality |
|  |  | Closed | Open |  | Risk of bias^a^ | Inconsistency | Indirectness | Imprecision | Publication bias |  |
| Rectal bleeding | 6 [12–17] | 650 | 603 | 0.57 [0.35-0.94] | Serious | Not serious | No indirectness | No imprecision | NA | Moderate |
| Operative time | 5 [12–15, 17] | 550 | 533 | 0.15 [0.03-0.28] | Serious | Not serious | No indirectness | No imprecision | NA | Moderate |
| Re-admission | 6 [12–17] | 650 | 603 | 0.34 [0.16-0.76] | Serious | Serious | No indirectness | Serious | NA | Low |
| Re-intervention | 3 [13, 15, 17] | 274 | 393 | 3.79 [1.24-11.57] | Serious | Not serious | No indirectness | Very serious | NA | Low |
| Overall postoperative morbidity | 4 [14–17] | 589 | 546 | 0.53 [0.39-0.73] | Serious | Serious | No indirectness | No imprecision | NA | Low |
| CI: Confidence interval, NA: Not applicable, OR: Odds ratio, SMD: Standardized mean difference  ^a^ Risk of bias assessed using the ROBINS-I tool | | | | | | | | | | |
